# Spatial Vision in *Bombus terrestris*

**DOI:** 10.3389/fnbeh.2016.00017

**Published:** 2016-02-15

**Authors:** Aravin Chakravarthi, Emily Baird, Marie Dacke, Almut Kelber

**Affiliations:** Department of Biology, Lund UniversityLund, Sweden

**Keywords:** spatial resolution, contrast sensitivity, insect vision, spatial vision, hymenoptera, bumblebees, dual choice test

## Abstract

*Bombus terrestris* is one of the most commonly used insect models to investigate visually guided behavior and spatial vision in particular. Two fundamental measures of spatial vision are spatial resolution and contrast sensitivity. In this study, we report the threshold of spatial resolution in *B. terrestris* and characterize the contrast sensitivity function of the bumblebee visual system for a dual choice discrimination task. We trained bumblebees in a Y-maze experimental set-up to associate a vertical sinusoidal grating with a sucrose reward, and a horizontal grating with absence of a reward. Using a logistic psychometric function, we estimated a resolution threshold of 0.21 cycles deg^−1^ of visual angle. This resolution is in the same range but slightly lower than that found in honeybees (*Apis mellifera* and *A. cerana*) and another bumblebee species (*B. impatiens*). We also found that the contrast sensitivity of *B. terrestris* was 1.57 for the spatial frequency 0.090 cycles deg^−1^ and 1.26 for 0.18 cycles deg^−1^.

## Introduction

Since the publication of Karl von Frisch's findings on the visual capacities of honeybees at the beginning of last century (Frisch, 1914), a plethora of literature has emerged on the visually guided behaviors of bees, including navigation, foraging and homing (see, for instance; Avarguès-Weber et al., 2011; Srinivasan, 2011). Bees extract visual information from their environment with a remarkable degree of accuracy, they perceive not only color and achromatic contrast, but also other properties such as spatial frequency and contour orientation (Srinivasan and Lehrer, 1988; van Hateren et al., 1990; Lehrer, 1998; Srinivasan and Zhang, 2004).

The primary basis of spatial vision is spatial resolution, that is, the ability to resolve fine spatial details (De Valois and De Valois, 1990). Spatial resolution has been determined in many species of bees using several different methods; anatomical, optical and behavioral (e.g., Hecht and Wolf, 1929; Srinivasan and Lehrer, 1988; Macuda et al., 2001; Spaethe and Chittka, 2003; Somanathan et al., 2009; Zhang et al., 2014). However, in almost all these cases, spatial resolution has been estimated for patterns with contrasts exceeding 80%. Studies on birds, primates and other taxa demonstrate, that it is more informative to estimate spatial resolution for a broader range of contrasts (Lind and Kelber, 2011). This is because the fine details in the signal are lost if there is low contrast between the elements (Snyder et al., 1977). A comprehensive and reliable way to estimate spatial vision is to characterize the spatial contrast sensitivity function, which relates contrast sensitivity and spatial resolution. This can be achieved behaviorally in bumblebees by finding their contrast thresholds for gratings over a broad range of spatial frequencies.

Amongst the hymenoptera, honeybees have traditionally been used as a standard model for understanding visually guided behaviors (e.g., Lehrer, 1998; Srinivasan and Zhang, 2004; Avarguès-Weber et al., 2011). However, in recent years, studies in this area have started to focus also on another species of the same order, the bumblebee *Bombus terrestris* (e.g., Raine and Chittka, 2007; Skorupski et al., 2007; Ings et al., 2012). *B. terrestris* rely on visual signals for a variety of behaviors that are crucial for their evolutionary success including color discrimination (e.g., Dyer and Chittka, 2004), shape perception and visual generalizations (Ings et al., 2012). They can associate visual cues also with a pollen reward (Nicholls and Hempel de Ibarra, 2014). All of these visually guided behaviors require some degree of spatial vision, but up until now, only single object resolution has been investigated in *B. terrestris* (e.g., Spaethe and Chittka, 2003). To truly understand spatial resolution and thus visually guided behaviors in *B. terrestris*, it is necessary to estimate their spatial resolution and contrast sensitivity, neither of which have been investigated to date. The aim of this current study is therefore to characterize the spatial resolution and contrast sensitivity of *B. terrestris* using a dual-choice discrimination task. This is not only the first investigation of its kind in bumblebees, but also represents the first attempt to use such a method to characterize contrast sensitivity in an insect species.

## Materials and methods

### Experimental apparatus and visual stimuli

**Figure 1:**
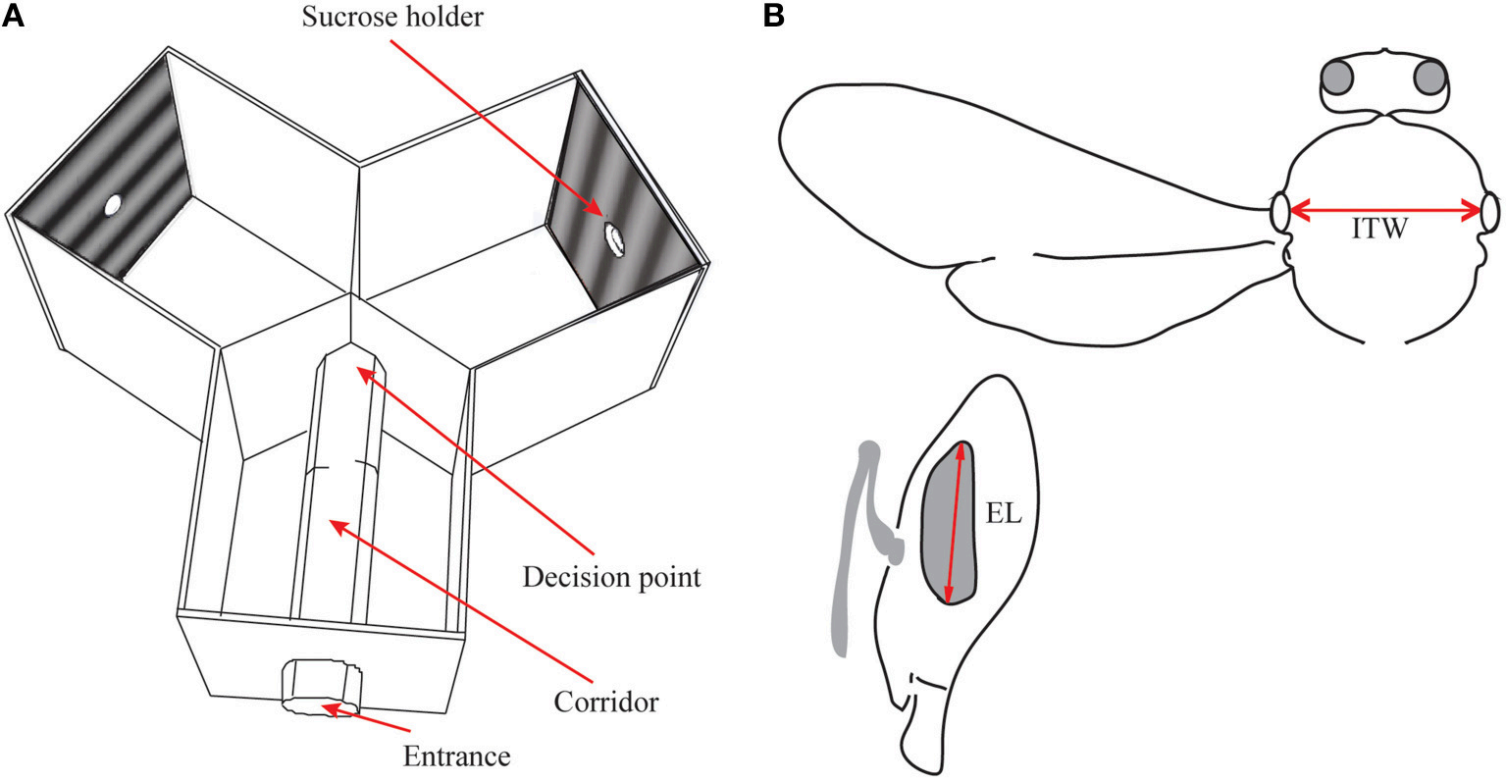
**(A)** Schematic diagram of the Y-maze apparatus used for the behavioral experiments. The bees entered the entrance arm and walked to the decision point, from where they flew towards the sucrose holder at the end of one of the arms. **(B)** Morphometrics used (see Spaethe and Chittka, 2003); dorso-ventral eye length (EL) and intertegular width (ITW) are measured as indicated by red arrows.

Colonies of *Bombus terrestris* were obtained from a commercial supplier (Koppert, UK) and placed in an indoor flight cage (2.2 m high, 1.7 m wide). The bees were exposed to a 9:15 h L:D cycle including 30 min long dawn and dusk periods with a maximum illuminance of 500 lux (BIOLUX, OSRAM GmbH, Munich, Germany) as measured from inside the testing apparatus using a photometer (Hagner ScreenMaster, B. Hagner, Solna, Sweden). The conditions inside the cage were kept relatively constant with a temperature of 25°C and 35–45% relative humidity. A netting wall divided the flight cage into two segments, a 190 cm long segment that housed the bumblebee colony and a smaller 100 cm long compartment where the transparent plexiglass Y-maze (Figure 1A) was placed on a platform, 75 cm above the floor. The free-flying bees could access the experimental compartment through a hole of 30 mm diameter.

The Y-maze had three identical arms (20 cm high, wide and long), with a 30 mm diameter clear plexiglass tube inserted in the entrance arm that opened into the two other arms of the maze (Figure 1A). As the bees were sitting on the narrow floor of the 30 mm diameter tube, their decision point was well defined. The bees learned to walk through this tube to reach the decision point at its end. At this point, the two visual stimuli placed in the arms of the Y-maze became visible and the bees could choose a stimulus to fly toward. The bees had to fly 26.5 cm from the decision point to reach the reward. By forcing the bees to make their decision in a stationary position at the end of the tube we ensured that they always made their decision at a set distance from the stimuli, allowing us to accurately calculate the spatial properties of the pattern at that point. A gating system in this entrance tube enabled us to test bees individually.

Each stimulus was mounted onto a 20 cm × 20 cm Plexiglas sheet, with a central hole connected to a detachable 100 mm long tube with 30 mm diameter, through which the bees could walk to receive a drop of 40% sucrose solution, presented through a syringe. The stimuli were achromatic sinusoidal gratings (printed on Canon Pro9000) of 6.6, 5, 2.5, 1.53, 1.25, 1.0, and 0.5 cm pattern wavelength, appearing as spatial frequencies of 0.069, 0.090, 0.18, 0.29, 0.36, 0.45, and 0.89 cycles deg^−1^ of visual angle at the decision point. The maximum (*I*_*Max*_) and minimum intensities (*I*_*Min*_) of these gratings were measured using a photometer (Hagner ScreenMaster, B. Hagner, Solna, Sweden) and the Michelson contrasts (MC) were calculated as,

(1) $$\begin{array}{r} MC=\frac{I_{Max}+\;I_{Min}}{I_{Max}-\;I_{Min}}. \end{array}$$

For contrast sensitivity experiments, we used gratings of 0.090 cycles deg^−1^ and 0.18 cycles deg^−1^ with Michelson contrasts of 89, 68, 54, 39, and 22%.

### Training procedure

Bees from 16 colonies were trained and tested over a period of 14 months. To motivate the naïve bees to forage in the Y-maze, we first removed the tubular corridor from the Y-maze and placed a vertically oriented sinusoidal grating of 0.090 cycles deg^−1^ in the entrance arm of the Y-maze. The bees could access the reward (40% sucrose solution in a cotton wick) by landing on the feeder tube inserted in the center of the pattern, and crawling through to its end. As the bees started to forage at the feeder, they were individually marked with plastic number plates on their thorax.

Next, we trained the bees to enter through the plastic tube in the entrance arm and to discriminate between a vertically and a horizontally oriented grating of 0.090 cycles deg^−1^. A drop of 40% sucrose solution was presented (in a detachable tube) behind the vertically oriented grating while the horizontally oriented grating presented no reward. The position of the rewarded and unrewarded stimulus was changed pseudo-randomly in order to avoid any side biases. During training, a choice of a bee was recorded only when the bee landed on the tube at the center of one of the two patterns without any backward flight loops or re-entry attempts from the decision point. If a bee reached the minimum learning criterion (choose the rewarded vertical grating significantly more often than chance (*p* < 0.05, binomial test) in a minimum of 10 and a maximum of 25 training trials) it was tested as described below. In total, 34 bees from 16 hives qualified to be tested. The tubes that presented either a reward or no reward could be easily detached. After every trial, the bee was released to the flight cage and the tubes were cleaned in ethanol. The position in the gratings was never changed, instead gratings were rotated by 90°. Thus, any possible scent marking on the grating, would be similar on both sides of the Y-maze.

### Testing procedure

For estimates of spatial resolution and contrast sensitivity, 20 choices per bee were recorded for 15 pairs of a vertically and a horizontally oriented grating of the same spatial frequency and contrast (seven different spatial frequencies, two of them at five different contrasts, see above).

For statistical analysis, we pooled the data from all animals tested at each spatial frequency and transformed the spatial frequencies into spatial wavelengths. To determine the threshold of spatial resolution and contrast sensitivity, a logistic psychometric function was fitted to these data:

(2) $$\begin{array}{r} \psi \left(x\right)=\gamma +\left(1-\;\gamma -\;\varepsilon \right){\left(1+e^{\left(\frac{a-x}{b}\right)}\right)}^{-1}, \end{array}$$

where ψ (*x*) is the proportion of correct choices at any spatial frequency or contrasts, γ is the lower asymptote that was fixed to 0.5, ε is the difference between the upper asymptote and 1.0 that was limited to between 0.0 and 0.2, and *a* and *b* are unrestricted parameters that determine the slope position and steepness of the fit (Wichmann and Hill, 2001a,b). A maximum likelihood method was used to fit the psychometric function to the behavioral data set, and evaluated the robustness of the psychometric fits by resampling the measured data using non-parametric bootstrapping. Bootstrapping was performed in Matlab using the program Palamedes (www.palamedestoolbox.org; Prins and Kingdom, 2009). The contrast sensitivity data were fitted using double exponential function which is commonly used for such data (Uhlrich et al., 1981).

### Morphometry

The bees used in the behavioral experiments were collected and killed (by exposing them to −20°C), harnessed in a plasticine holder, and measured for body size (intertegular width and eye length, see Spaethe and Chittka, 2003; Figure 1B) under a stereo microscope. Earlier studies in bumblebees have shown a linear relationship between body size and object resolution (Spaethe and Chittka, 2003). To test if the results of our experiments were influenced by the size of the bees tested for each spatial frequency, we compared if eye length and spatial frequency conditions had any relationship. To account for repeated measures, we used a mixed-effects model to fit the data. This model was tested against a random effects model (effect of individual only) with a Chi-square test and by a comparison of the Akaike Information Criterion (AIC; Akaike, 1974). The model with the lower AIC is the better fit model.

## Results

### Estimation of the spatial resolution

**Figure 2:**
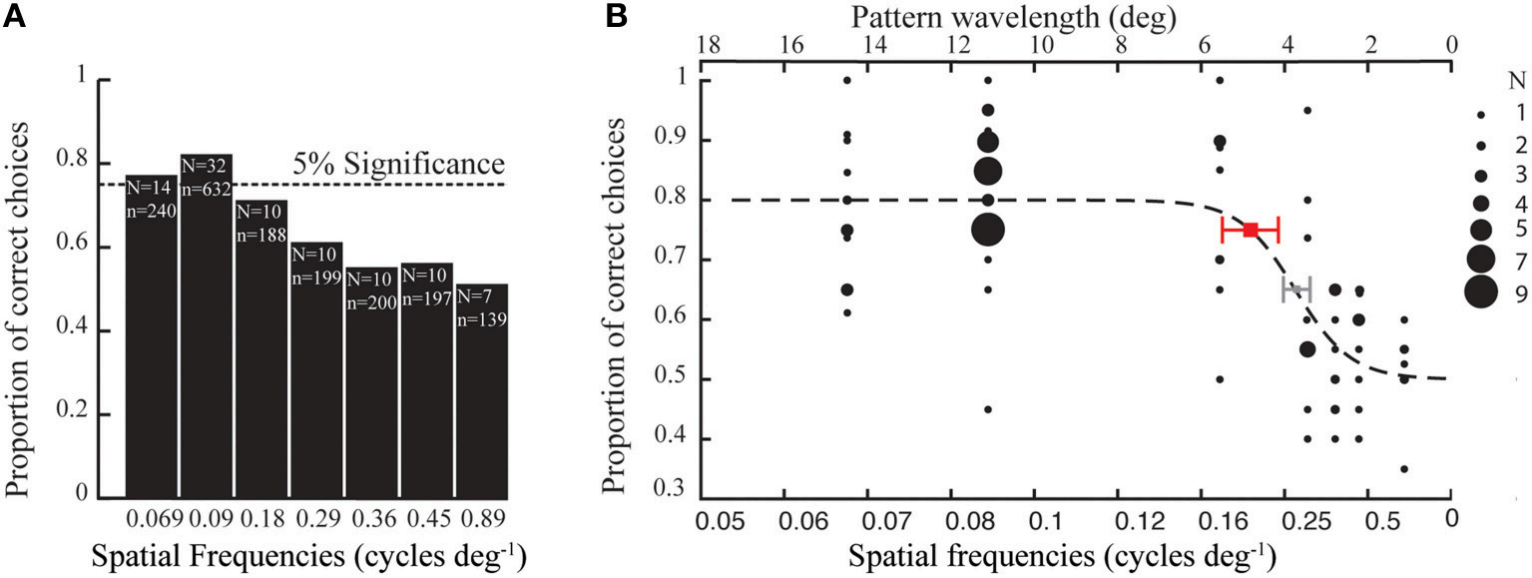
**(A)** Average proportion of correct choices made by *Bombus terrestris* in tests with gratings of different spatial frequencies (1795 choices by 34 bees). Dotted line at 0.75 indicates the proportion of correct choices above the threshold (binomial test, *p* < 0.05), N indicates the number of bees tested and n indicates the number of choices made for each spatial frequency. **(B)** Each black filled circle denotes the proportion of correct choices performed by a certain number of individuals (see inset for details). The red and the gray square denote the thresholds at 0.75 and 0.65 proportion of correct choices, respectively, which is interpolated from the fitted (dotted line) logistic function (see Materials and Methods). The error bar indicates the 95% confidence interval of the threshold.

In the first set of experiments, the bumblebees (*Bombus terrestris)* were presented with high contrast gratings (87%) of seven different spatial frequencies (see methods). The proportion of correct choices out of 20 choices were averaged for all bees (34 bees) tested for each spatial frequency (Figure 2A). A logistic function was fitted to the data, with a threshold value at a proportion of 0.75 correct choices (binomial test, *p* < 0.05). From this, we determined that the spatial resolution threshold of the tested bees was 4.8° (Figure 2B), which is equivalent to a spatial frequency of 0.21 cycles deg^−1^. Thus, of the seven spatial frequencies tested to estimate the spatial resolution of *B. terrestris*, only the three lowest frequencies could be reliably resolved; 6.6, 5.0, and 2.5 cm pattern wavelength appearing as 0.069, 0.090, and 0.18 cycles deg^−1^ (Figure 2B).

### Contrast threshold and contrast sensitivity function

**Figure 3:**
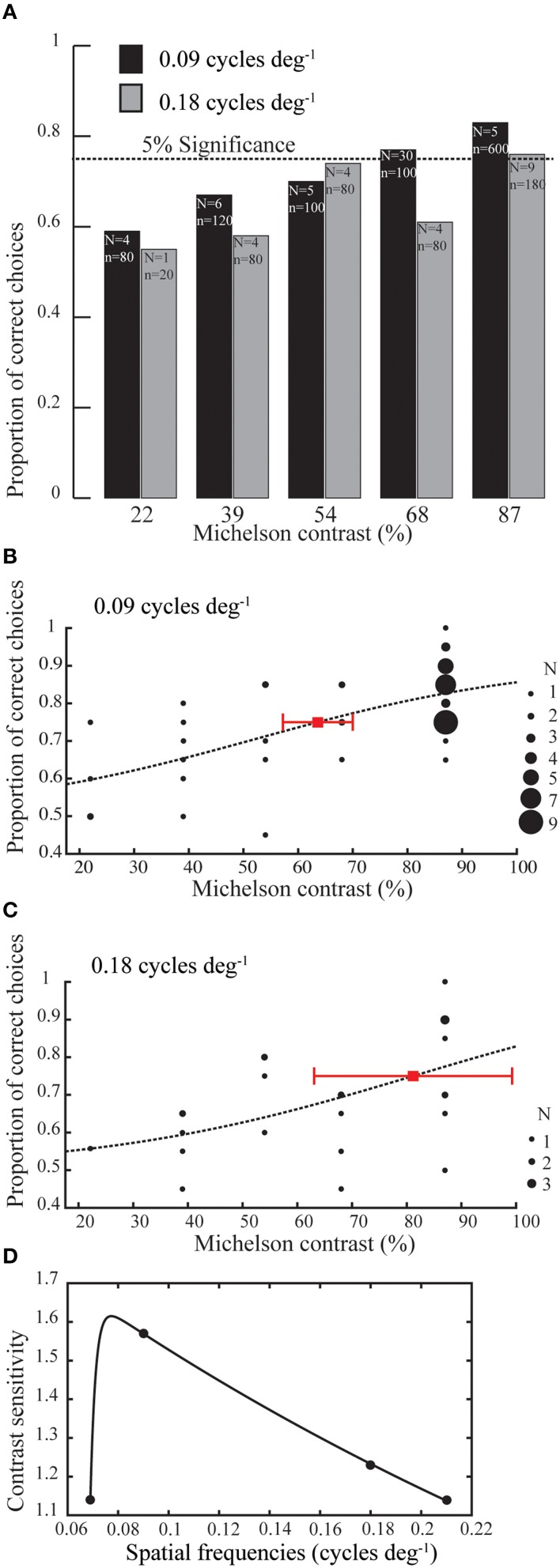
**(A)** Average proportion of correct choices made by *Bombus terrestris* for gratings of 0.090 (black bars) and 0.18 cycles deg^−1^ (gray bars) for a range of Michelson Contrasts. N indicates the number of bees tested and n indicates the number of choices made for each spatial frequency. **(B,C)** Behaviorally determined contrast threshold for grating of 0.090 (1000 choices) and 0.18 (440 choices) cycles deg^−1^ respectively. Each black filled circle denotes the proportion of correct choices performed by a certain number of individuals (varies with the diameter of the filled circle, see inset for details). Red squares in **(B,C)** denotes the contrast thresholds at 0.75 proportion correct choices that are interpolated from the fitted (dotted line) logistic function (see methods). Contrast thresholds of 63.6 and 81% Michelson contrast for 0.090 and 0.18 cycles deg^−1^ are interpolated at the threshold (red squares). Error bars indicate 95% confidence intervals of the thresholds. **(D)** Contrast sensitivity function of *Bombus terrestris*. Contrast sensitivity is expressed as the inverse of the contrast sensitivity threshold. We used a double exponential fit for the data that shows a peak of 1.61 (62% contrast threshold) for 0.078 cycles deg^−1^.

To test the contrast thresholds of the bees, we presented the bees with two of the spatial frequencies that could be reliably resolved at high contrast (0.090 cycles deg^−1^, 0.18 cycles deg^−1^) at four lower contrasts (68, 54 39, and 22%). Figure 3A shows the average proportions of correct choices for all contrasts tested. Again, a logistic function was fitted to the data to determine the contrast threshold for each of the spatial frequencies (Figures 3B,C). The contrast threshold for 0.090 cycles deg^−1^ and 0.18 cycles deg^−1^ was found at 63.6 and 81% Michelson contrast respectively. Since the proportion of correct choices for the high contrast (87%) grating of 0.069 cycles deg^−1^ (0.76) was very close to the threshold of 0.75, we set 87% as the contrast threshold for 0.069 cycles deg^−1^. A prediction of 87% contrast threshold was also made for the spatial resolution threshold (0.21 cycles deg^−1^). This results in a contrast sensitivity of 1.15 for both of these spatial frequencies. The resulting contrast sensitivity function of *B. terrestris* is shown in Figure 3D, where a double exponential fit has been applied to the data.

### Morphometric measurements of bees tested with different spatial frequencies

**Figure 4:**
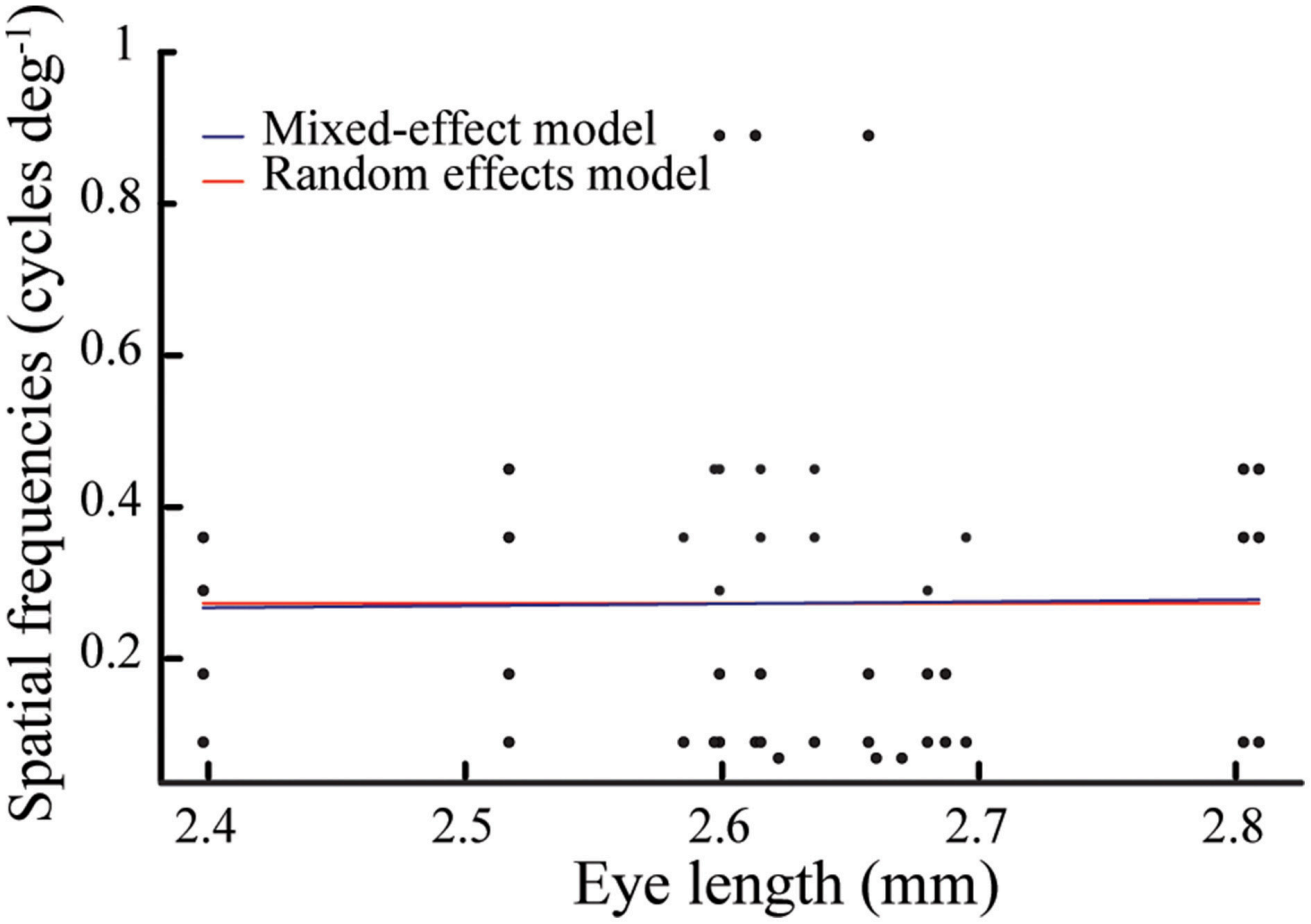
**Eye length of bees (*N* = 18, *n* = 47) tested at seven spatial frequencies (0.069, 0.090, 0.18, 0.29, 0.36, 0.45, and 0.89 cycles deg^−1^)**. Note that most bees were tested with more than one spatial frequency. Assuming that eye length is the most relevant measure of body size, we fitted a mixed-effects model (blue line) to our data to compare if eye length and spatial frequency conditions had any relationship. This model was not a significantly better fit than a null model (red line), which reveals that eye length and spatial frequency tested had no relationship (for statistical details, see Results).

We could perform morphometric measurements on 18 of the 34 tested bees; we measured their intertegular width, which ranged from 3.16 to 4.34 mm, with an average of 3.89 ± 0.28 mm (mean ± standard deviation). The eye length ranged from 2.38 to 2.81 mm, with an average of 2.64 mm ± 0.10 mm. In contrast to earlier studies (Spaethe and Chittka, 2003), the correlation between these two measurements was not statistically significant (*r* = 0.46, *p* = 0.06), which is possibly due to the small sample size and the narrow size range of the bees that visited the Y-maze in this study. Assuming that eye length is the most relevant measure of body size for our study, we fitted a mixed-effects model to our data to test the relationship of spatial frequency condition to eye length. This model was not a significantly better fit than a random effects model, which reveals that eye length and spatial frequency tested had no relationship [Δ deviance = 0.007, *df* = 1, *p* = 0.93; AIC (mixed-effects model) = 1.81 vs. AIC (random effects model) = −0.69; Figure 4].

## Discussion

Here, we used a dual choice discrimination assay to investigate two basic aspects of spatial vision in *Bombus terrestris*; the threshold of behavioral spatial resolution for achromatic sinusoidal gratings and the contrast sensitivity function.

### The spatial resolution threshold of *B. terrestris* is 0.21 cycles deg^−1^

Here, we determined the spatial resolution threshold of *B. terrestris* as 0.21 cycles deg^−1^ or 4.8° spatial wavelength for the discrimination of horizontally vs. vertically oriented sinusoidal grating patterns (Figure 2B). An earlier study on single object resolution has revealed that *B. terrestris* can detect yellow targets on a white background subtending a minimum visual angle between 3.5° and 7°, depending on the body size of the bee (Spaethe and Chittka, 2003). Bees comparable in size to the ones tested in our experiments (2.64 ± 0.10 mm eye length) could detect targets of 6.3°. Interestingly, in two later studies (Dyer et al., 2008; Wertlen et al., 2008), *B. terrestris* was even found to detect yellow targets subtending a visual angle as small as 2.3°. Unfortunately, these latter studies did not report the body size of the tested bees. Nevertheless, the smallest detectable target size presented in these studies lies in a similar range as half the minimal detectable grating wavelength (4.8°) determined in our experiments, with each grating stripe occupying 2.4°.

For similarly sized bees as the ones tested here (with a mean intertegular width of 3.89 mm), Spaethe and Chittka (2003) measured the smallest interommatidial angles to be approximately 0.9° in the vertical, and 2.3° in the horizontal direction. From this we can get an estimate of the spatial resolution of the eye using Equation (3):

(3) $$\begin{array}{r} \nu_{s}=\frac{1}{2△\phi }, \end{array}$$

where the minimum spatial frequency (ν_*s*_) resolvable by two adjacent receptors is the inverse of twice the corresponding interommatidial angle △ϕ (Wehner, 1981). The spatial resolution limit estimated from these measurements, between 0.55 and 0.21 cycles deg^−1^, corresponds well to the spatial resolution threshold *of B. terrestris* of 0.21 cycles deg^−1^ presented here.

### Spatial resolution of other bee species

A dual choice discrimination task, similar to the task presented here, has been used to estimate the spatial resolution threshold in other species of bees; 0.26 cycles deg^−1^ (or a pattern wavelength of 3.8°) for the European honeybee *Apis mellifera* (Srinivasan and Lehrer, 1988) and 0.26–0.35 cycles deg^−1^ for the Eastern honeybee, *A. cerana* (Zhang et al., 2014). This suggests that these two species of honeybee have a slightly higher spatial resolution threshold than *B. terrestris*. In several studies, European honeybees were found to detect single objects of a minimum angle of 5° (Giurfa et al., 1996, 1997; Hempel de Ibarra et al., 2001, 2002). This is a larger angle than half the wavelength of gratings that bees of this species could resolve.

The resolution threshold of another bumblebee species, *B. impatiens* was found to lie around 0.35 cycles deg^−1^ (Macuda et al., 2001), indicating that these bees also have higher spatial resolution than *B. terrestris*. However, this study (Macuda et al., 2001) set the resolution threshold to a proportion of 0.65 correct choices rather than the 0.75 used in this present study. A threshold criterion of 0.75 correct choices would have resulted in a spatial resolution threshold of approximately 0.26 cycles deg^−1^ for *B. impatiens*, which is similar to the threshold defined for *B. terrestris* (gray bar in Figure 2B).

The spatial resolution of *B. impatiens* has also been estimated in a different behavioral context; the centering response of free flying bees (Dyhr and Higgins, 2010). In their study, the resolution threshold was determined from the ability of the bee to center between one wall carrying black and white gratings of different spatial frequencies, and a uniformly gray wall. In this task, *B. impatiens* fail to resolve spatial frequencies higher than 0.14 cycles deg^−1^. However, this estimate of spatial resolution depends upon where in the visual field the bees are expected to measure optic flow for position control. This is because the perceived spatial frequency of a pattern lining the walls of a corridor will vary with the angular position at which it is being measured. Given that *B. terrestris* can measure image motion at much lower viewing angles than 90° (Baird et al., 2010; Linander et al., 2015) it is very likely that the spatial frequency resolution of *B. impatiens* is somewhat higher than the 0.14 cycles deg^−1^ calculated for a viewing angle of 90°. Another possible explanation for the difference between the spatial resolution limits in *B. impatiens* measured in the dual choice discrimination task and the centering task is that the motion detection pathway has a different spatial resolution threshold than the object detection pathway. Experiments with *B. terrestris* are planned to test this hypothesis.

### Spatial resolution of other insect species

Apart from bees, spatial resolution of single object detection has also been investigated in several other insects with behavioral tests. Takeuchi et al. (2006) used a dual choice experiment similar to the one we have used in this study to find the resolution limit of the butterfly *Papilio xuthus*. *P. xuthus* detected blue, green and red circular discs subtending visual angles exceeding 1.18°, 1.53°, and 0.96° respectively. Assuming that the angle subtended by each disc as equivalent to the angle subtended by half the grating wavelength, spatial resolution expressed in cycles deg^−1^ for blue, green and red discs would be 0.42, 0.32, and 0.52 cycles deg^−1^ respectively. A similar experimental approach has also been employed to estimate the spatial resolution of three species of psyllids (Farnier et al., 2015). Farnier et al. (2015) estimated resolution thresholds of 8.7° of visual angle for a yellow disc by *Ctenarytaina eucalypti*, and thresholds of 6.8 and 6.3° of visual angle for a red disc in *Anoeconeossa bundoorensis* and *Glycaspis brimlecombei* respectively. This translates into a resolution range of 0.05–0.08 cycles deg^−1^ in these psyllids. In a target pursuit task by the praying mantis, *Euchomenella macrops*, Prete et al. (2012) found that the resolution threshold was about 0.9° of visual angle. Wardill et al. (2015) estimated the spatial resolution for moving targets to lie between 0.5 and 0.9° of visual angle in killer flies *Coenosia attenuate* performing a similar task.

The single object resolution on these insect species can be compared to that of *B. terrestris* (Spaethe and Chittka, 2003), which ranged between 3.5° and 7° of visual angle. However, to our knowledge, no experiments testing resolution of stationary gratings have been done in other insect species.

### The contrast sensitivity of *Bombus terrestris* is surprisingly low

In our experiments, we found a rather low contrast sensitivity of 1.57 (63.6% contrast) at 0.090 cycles deg^−1^ and 1.23 (81% contrast) at 0.18 cycles deg^−1^ in *B. terrestris*, and a peak sensitivity of 1.61 (62% contrast) for 0.078 cycles deg^−1^ (Figure 3D). In contrast, *Apis mellifera* are able to discriminate vertical from horizontal gratings at 0.1 cycles deg^−1^ at only 8% contrast (Srinivasan and Lehrer, 1988), equivalent to a contrast sensitivity of 12.5 or more. There are several possible reasons that could explain this difference.

Firstly, contrast sensitivity is luminance-dependent and, as light intensity decreases, the peak of the contrast sensitivity begins to drop, shifting the peak sensitivity toward lower spatial frequencies (e.g., De Valois and De Valois, 1990). The illumination conditions in our experiment and those in the experiments of Srinivasan and Lehrer (1988) were different. Srinivasan and Lehrer (1988) tested bees in a room with natural daylight coming in through a large window. We tested bees in 500 lux, which is approximately two orders of magnitude lower than the brightest daylight conditions, but still well within the functional range under which they forage (Reber et al., 2015). The large difference in contrast sensitivity between these two studies may thus partly be a consequence of the difference in light intensities. Luminance-dependence of spatial vision in bees remains to be investigated.

Another potential explanation for the differences between the measured contrast sensitivities of honeybees and bumblebees is that, in their experiments, Srinivasan and Lehrer (1988) used square wave gratings whereas we used sinusoidal gratings. Square wave gratings have prominent edges between the stripes of high and low intensities, and it is possible that lateral inhibition between receptors looking at both sides of this edge will be stronger than in an experiment with sinusoidal gratings without prominent edges. Such an edge-detection mechanism may have contributed to the differences between our results and those obtained by Srinivasan and Lehrer (1988). In their tests of centering response, also found that *B. impatiens*, Dyhr and Higgins (2010) also found differences in the response to sinusoidal and square wave gratings.

It has also been shown that the conditioning procedure can affect the learning of visual targets in bees (reviewed by Avarguès-Weber and Giurfa, 2014). Our results are based on non-aversive differential conditioning. Bumblebees that were rewarded when choosing the correct stimulus, and punished when choosing the incorrect stimulus in a dual choice test were able to discriminate smaller color differences, compared to bees that only received a reward (Dyer and Chittka, 2004). Maybe punishment would have improved the performance of the bees in our experiment as well. Moreover, we tested the bees for randomly selected low contrast gratings after specifically training them to a low frequency high contrast sinusoidal grating. It is possible that bees did not generalize between the high contrast grating and the low contrast gratings. This approach may also have contributed the low sensitivity determined in our tests. To reach a comparative understanding of spatial resolution and contrast sensitivity specific to certain visual tasks and specific visual pathways of different species of bees, additional detailed and directly comparable investigations will be required.

Finally, unlike Srinivasan and Lehrer's (1988) honeybees, which had the freedom to make side-wise flight loops in the decision chamber while taking a decision, the bees in our experiment took decisions from a restricted decision point. Thus, for the honeybees, the stimulus appeared to be in motion, while for the bumblebees, the stimulus appeared stationary. From birds we know that the contrast sensitivity is higher for moving stimuli (Haller et al., 2014). As far as we know contrast sensitivity for stationary objects has not been tested in other insects.

## Author contributions

AK, MD, EB, and AC conceptualized the study. AC performed the experiments and analyzed the data. AC and AK interpreted the data. AC wrote the manuscript with contributions from AK, MD, and EB.

### Conflict of interest statement

The authors declare that the research was conducted in the absence of any commercial or financial relationships that could be construed as a potential conflict of interest.
